# Comparison of the efficacy and safety profiles of a pelubiprofen versus celecoxib in patients with rheumatoid arthritis: a 6-week, multicenter, randomized, double-blind, phase III, non-inferiority clinical trial

**DOI:** 10.1186/1471-2474-15-375

**Published:** 2014-11-18

**Authors:** In Ah Choi, Han-Joo Baek, Chul-Soo Cho, Yeon-Ah Lee, Won Tae Chung, Young Eun Park, Yun Jong Lee, Yong-Beom Park, Jisoo Lee, Shin-Seok Lee, Wan-Hee Yoo, Jung-Soo Song, Seong Wook Kang, Hyun Ah Kim, Yeong Wook Song

**Affiliations:** Division of Rheumatology, Department of Internal Medicine, Seoul National University Hospital, 101 Daehak-ro, Jongno-gu, Seoul 110-744 South Korea; Division of Rheumatology, Department of Internal Medicine, Gachon Medical School Gil Medical Center, Incheon, South Korea; Division of Rheumatology, Department of Internal Medicine, Yeouido St. Mary's Hospital Catholic University Medical College, Seoul, South Korea; Division of Rheumatology, Department of Internal Medicine, Kyung-Hee University Medical Center, Seoul, South Korea; Division of Rheumatology, Department of Internal Medicine, Dong-A University Hospital, Pusan, South Korea; Division of Rheumatology, Department of Internal Medicine, Pusan National University Hospital, Pusan, South Korea; Division of Rheumatology, Department of Internal Medicine, Seoul National University Bundang Hospital, Seongnam, South Korea; Division of Rheumatology, Department of Internal Medicine, Severance Hospital, Seoul, South Korea; Division of Rheumatology, Department of Internal Medicine, Ewha Women's University Mokdong Hospital, Seoul, South Korea; Division of Rheumatology, Department of Internal Medicine, Chonnam, National University Hospital, Gwangju, South Korea; Division of Rheumatology, Department of Internal Medicine, Chonbuk National University Hospital, Jeonju, South Korea; Division of Rheumatology, Department of Internal Medicine, Chung-Ang University Hospital, Seoul, South Korea; Division of Rheumatology, Department of Internal Medicine, Chungnam National University Hospital, Daejeon, South Korea; Division of Rheumatology, Department of Internal Medicine, Hallym University Sacred Heart Hospital, Anyang, South Korea; Department of Molecular Medicine and Biopharmaceutical Sciences, Graduate School of Convergence Science and Technology, and College of Medicine, Medical Research Center, Seoul National University, Seoul, South Korea

**Keywords:** Rheumatoid arthritis, Pelubiprofen, Celecoxib, Non-inferiority

## Abstract

**Background:**

Pelubiprofen is a prodrug of 2-arylpropionic acid with relatively selective effects on cyclooxygenase-2 activity. The aim of this study was to compare the efficacy and safety profiles of pelubiprofen with those of celecoxib in patients with rheumatoid arthritis.

**Methods:**

This was a 6-week, multicenter, randomized, double-blind, double-dummy, parallel-group, phase III, non-inferiority clinical trial. The primary end point was non-inferiority of pain decrease from baseline to week-6 as determined using a 100 mm pain visual analog scale (VAS). Pelubiprofen was considered non-inferior to celecoxib if the lower limit of the 97.5% confidence interval for treatment difference [(pain reduction in pelubiprofen group) – (pain reduction in celecoxib group)] was more than −10 mm. The secondary end points were as follows: non-inferiority of (1) reduction of Korean health assessment questionnaire (KHAQ) score; (2) decreased duration of morning stiffness; and (3) decrease in the frequency and total dose of rescue drugs after 6 weeks of treatment.

**Results:**

Seventy-seven patients in the pelubiprofen group and 68 patients in the celecoxib group started the study medication. Pelubiprofen was non-inferior to celecoxib with regard to reduction in VAS pain severity (difference, mean ± SD 5.0 ± 20.1; 97.5% CI, −2.3 to ∞). Pelubiprofen was also non-inferior to celecoxib in terms of the secondary end points, such as, decrease in KHAQ score (0.0 ± 0.5, 97.5% CI −0.2 to ∞), decrease in duration of morning stiffness (median 0.0 minute in both groups), and decrease in the frequency (0.7 ± 3.5, 97.5% CI −0.6 to ∞) and total amount (0.7 ± 3.6, 97.5% CI −0.6 to ∞) of rescue medication uses during the 6 week study period. Safety analysis revealed 31.2% patients in the pelubiprofen group and 20.6% patients in the celecoxib group experienced an adverse drug reaction (ADR). The frequency of gastrointestinal ADRs was 20.8 % and 8.8%, respectively.

**Conclusions:**

Pelubiprofen was found to be as effective as celecoxib at pain reduction and for relieving stiffness in RA patients. However, more patients in the pelubiprofen group experienced ADR and the frequency of gastrointestinal ADRs was higher in the pelubiprofen group. ClinialTrials.gov identifier: NCT01781702.

**Electronic supplementary material:**

The online version of this article (doi:10.1186/1471-2474-15-375) contains supplementary material, which is available to authorized users.

## Background

Rheumatoid arthritis (RA) is a chronic inflammatory arthritis and often results in joint damage and physical disability. Nonsteroidal anti-inflammatory drugs (NSAIDs) alleviate pain and stiffness and are widely used to control the symptoms of RA. Patients tend to take multiple medications including corticosteroids, NSAIDs, and disease-modifying anti-rheumatic drugs (DMARDs). However, the long-term use of NSAIDs is frequently limited by gastrointestinal (GI) adverse effects, such as, dyspepsia, abdominal pain, gastric ulcers, and bleeding [1, 2]. Cyclooxygenase (COX)-2 selective inhibitor was developed to improve this unsatisfactory profile of NSAIDs and its use is common in the elderly and in those who are at risk of GI bleeding, including patients with RA [3–5].

Pelubiprofen is a member of the 2-arylpropionic acid family, which is related structurally and pharmacologically to ibuprofen. Pelubiprofen is known to inhibit COX activity and the transforming growth factor-β activated kinase 1 - IκB kinase β - NF-κB pathway [6], and in clinical studies was found to have significant anti-inflammatory and analgesic effects [Gu-youn Kwon: Phase II and III clinical study report of pelubiprofen (DW-330), Daewon Pharm, data unpublished]. The Korean Ministry of Food and Drug Safety approved pelubiprofen for relieving the symptoms of osteoarthritis (OA) in 2007 and approved expanded indications for the relief of symptoms of back pain in 2010. This phase III trial was designed as a part of new drug application to expand the indications of pelubiprofen for relieving the symptoms of RA.

Pelubiprofen is believed to cause fewer GI adverse events than traditional NSAIDs because it is a prodrug. In addition, it has selective effects on COX-2 activity (COX-1/COX-2 ratio: 3.7) [6]. Therefore, we expected pelubiprofen to be useful at relieving the symptoms of RA in patients at high risk of an adverse GI event. The objective of this trial was to compare the efficacy and safety profiles of pelubiprofen with those of celecoxib in patients with moderate to severe RA. In the current study, we tested the hypothesis that pelubiprofen would be non-inferior to celecoxib in terms of pain reduction, improving quality of life, reducing morning stiffness and overall safety in patients with moderate to severe RA.

## Methods

### Patients

Participants were all Korean, aged 18 to 80 years, and taking NSAIDs for the treatment of RA. All satisfied the 1987 American College of Rheumatology (ACR) classification criteria of RA [7], and had a disease duration of over 3 months and an ACR functional class of I, II or III [8]. Furthermore, all had been taking stable doses of DMARDs, such as, methotrexate, sulfasalazine, hydroxychloroquine, or leflunomide for over 3 months at the screening visit and maintained these doses during the study period. A stable low dose of prednisolone at 10 mg or less per day over 4 weeks was permitted. On randomization, patients were required to have worsened pain, defined as an increase in pain visual analog scale (VAS) score at least 10 mm or of 20% from baseline during the washout period, and to have moderate or severe arthritis, which was defined as a disease activity score of 28 (DAS28) ≥3.2.

Patients were excluded if they had a history of hypersensitivity to NSAIDs or a serious cardiovascular, liver, kidney or blood disease or another autoimmune disease. Those with a peptic ulcer or GI bleeding confirmed by endoscopy or radiography within 6 months of enrollment or who could not discontinue their GI medication, such as, H2 blocker, misoprostol or proton pump inhibitor were excluded. Patients were also excluded if they had been treated with intra-articular corticosteroid within 4 weeks prior to screening or had previously been administered biologic DMARDs, such as, infliximab, adalimumab, etanercept, anakinra, or abatacept within 6 months or rituximab within 1 year of enrollment. Pregnant women, breastfeeding women, and women of childbearing potential not using an appropriate method of contraception, such as, condoms, intrauterine devices, and oral contraceptives, were excluded.

### Study procedure

This was a 6-week, multicenter, randomized, double-blind, double-dummy, parallel-group, phase III, non-inferiority clinical trial conducted at 14 medical centers in Korea from October 2010 to October 2011. The study protocol was approved by the Institutional Review Boards at each of the participating institutes, which were; Seoul National University College of Medicine-Seoul National University Hospital Institutional Review Board (IRB), Gachon University Gil Medical Center IRB, The Catholic University of Korea, Yeouido St. Mary's Hospital Catholic IRB, Kyung-Hee University Hospital IRB, Dong-A University Hospital IRB, Pusan National University Hospital IRB, Severance Hospital IRB, Ewha Womans University Medical Center IRB, Chonnam National University Hospital IRB, Chonbuk National University Hospital IRB, Chung-Ang University Hospital IRB, Chungnam National University Hospital IRB, and Hallym University Sacred Heart Hospital IRB. Written informed consent was obtained from each patient before study enrollment. No important change to study methods was made after trial commencement.

Study medications and comparators were packed in identical appearance and consecutively numbered according to the allocation sequence. Random allocation sequence generated using SAS 9.1v software (SAS Institute, Cary, NC) by an independent statistician and was stratified by center with a 1:1 allocation using random block sizes of 4 and 6. After a 3–14 day washout period depending on the half-lives of NSAIDs, patients who experience pain worsening were assigned order numbers by investigators at each center and received the corresponding medication from pharmacists. A double-dummy design was implemented because the pelubiprofen tablet and celecoxib capsule differed in appearance and dosage. Patients in pelubiprofen group received a pelubiprofen 30 mg tablet three times daily and a celecoxib placebo capsule twice daily. Patients in celecoxib group received a 200 mg Celebrex® capsule twice daily and pelubiprofen placebo tablet three times daily. The allocation sequence was concealed from the researcher enrolling and assessing participants and who kept randomization envelopes. The sponsor and the principal investigators at each center were in charge of the envelopes, which were opaque, sealed, and not opened until trial completion. Blinding could only be broken in emergency situations for reasons of patient safety.

The drug administration period was 6 weeks. During weeks 0, 2 and 4, participants were given sufficient study medication to last until their next scheduled visit plus an additional amount to accommodate visits scheduled within the allowed visit variance (±4 days), and were asked to return all unused medication during visits on weeks 2, 4, and 6. Compliance with study medication was monitored by noting returned medication on case report forms. Rescue medication was allowed in the form of an acetaminophen extended-release (ER) 650 mg tablet. The total amount of rescue medication used was tracked using an accountability procedure.

### Efficacy and safety profiles

The primary end point was pain decrease from baseline to week 6 as determined using a 100 mm pain VAS. Patients specified general pain experienced during the previous 48 hours by indicating a position along a continuous line between the two end-points (0 mm = no pain and 100 mm = severest pain imaginable) under the supervision of the study investigator [9]. Changes in VAS pain from week 0 to 6 in the two groups were compared using the t-test and are presented with 97.5% confidence intervals (CI). We hypothesized that pelubiprofen was non-inferior to celecoxib if the lower limit of the 97.5% confidence interval for the treatment difference [(pain reduction in pelubiprofen group) – (pain reduction in celecoxib group)] was more than −10 mm. Secondary end points were as follows: (1) reduction of Korean health assessment questionnaire (KHAQ) score; (2) decreased duration of morning stiffness; and (3) decrease in the frequency and total dose of rescue drugs after 6 weeks of treatment.

Differences in the duration of morning stiffness between baseline and week 6 were calculated and presented in median. Duration of morning stiffness is reported in minutes and durations of more than 360 minutes are reported as 360 minutes. The KHAQ consisted of 20 items in 8 categories that addressed; (1) dressing and grooming, (2) arising, (3) eating, (4) walking, (5) hygiene, (6) reach, (7) grip, and (8) common daily activities. For each of these categories, patients reported the amount of difficulty experienced when performing 2 or 3 items using a 4 score: 0 = without any difficulty, 1 = with some difficulty, 2 = with much difficulty, and 3 = unable to do. A category score was determined using the highest score of the items in that category. The sum of all category scores was then divided by the number of categories answered to produce a single disability index score [10, 11]. Usages of rescue medication were assessed at baseline and on treatment weeks 2, 4, and 6 and analyzed for frequencies and amounts of acetaminophen ER 650 mg tablets used during the previous 2 weeks by repeated measure analysis of variance (ANOVA). All the other efficacy assessments were performed at baseline and week 6.

The investigators, who were blinded to treatment group identities, described and assessed all clinical and laboratory adverse events (AEs). Clinical AEs were evaluated by physical examination and general questioning at every visit, and laboratory AEs were evaluated by complete blood count, serum chemistry and urinalysis at screening and last visit (week 6). All AEs were categorized according to the likelihood of a causal relationship with the study drug, that is, as definitely related, probably related, possibly related, probably not related, definitely not related, or unknown [12]. Causal relationships of AEs were determined based on clinical judgment by the investigators. Adverse drug reactions (ADRs) were defined as AEs that were at least possibly related to study medications. Serious AEs and ADRs were defined as those associated with any of the following: death; an event associated with a high risk of mortality; an event requiring hospitalization; or the development of a permanent disability or congenital malformation.

### Statistical analyses

Baseline patient characteristics in the pelubiprofen and celecoxib study groups were analyzed. Continuous variables are expressed as means ± standard deviations (SD), and categorical variables as numbers and percentages.

For the sample size calculation, we referred to the report of Song et al. [13]. In this non-inferiority trial using celecoxib, reduction in 100 mm VAS pain was 17.87 mm and its SD was 19.06 mm. Based on the non-inferiority margin of −10 mm reported, pelubiprofen was considered non-inferior to celecoxib if the lower limit of the 97.5% CI of treatment difference [(pain reduction in the pelubiprofen group) – (pain reduction in the celecoxib group)] was more than −10 mm. With a 1-sided significance level of 0.025 and assuming a SD of 19.06 mm, a sample size of 58 patients per group was determined to provide a study power of 80%, and thus, 73 patients were deemed to be required given a 20% dropout rate. Secondary outcomes were also tested for non-inferiority using one-sided tests.

For the efficacy profile analysis, per protocol (PP) populations were used for the main analysis and intent-to-treat (ITT) populations were used for supplementary analysis with the input of any missed observations according to the last observation-carried-forward method.

The safety population included all patients who had received ≥1 dose of study medication after randomization and incidences of AEs and of ADRs in the study groups were compared using the χ^2^ test or Fisher’s exact test. The ITT population included all patients who had received ≥1 dose of study medication after randomization and were available for analysis of the primary end point. The PP population included only those patients who had completed the protocol at the end of the study period. Protocol violators and patients with <80% drug compliance were excluded from the PP analysis. Statistical analysis was conducted using SAS version 9.1 (SAS Institute Inc., Cary, North Carolina). Statistical significance was accepted for p values <0.05.

## Results

### Patient characteristics

**Figure 1:**
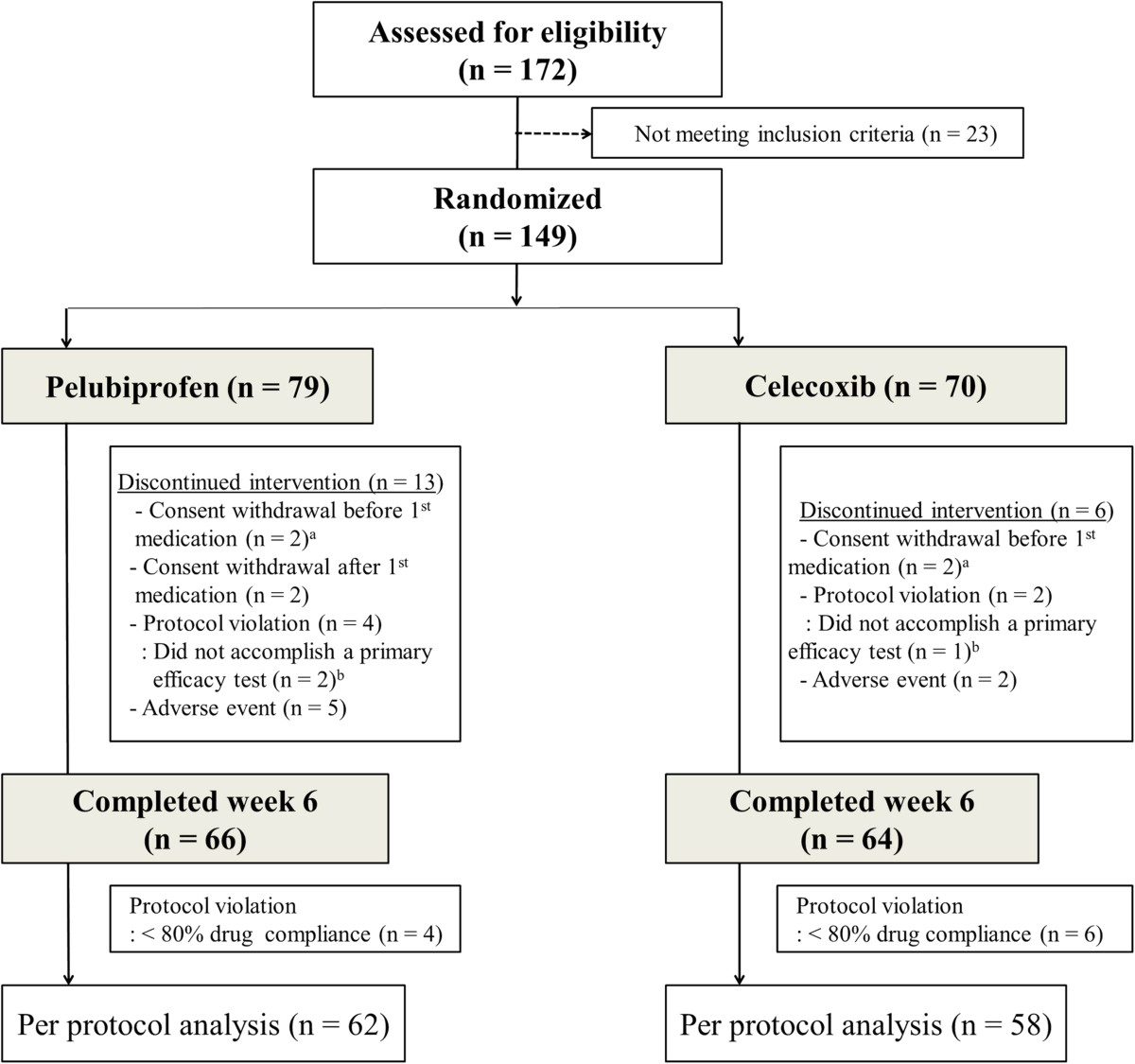
**Patient allocation, follow-up, and analysis in the study of pelubiprofen versus celecoxib for the patients with rheumatoid arthritis.** ^a^Excluded from safety analysis. ^a+b^Excluded from intention to treat analysis.

Of the 172 patients assessed for eligibility, 149 satisfied the inclusion/exclusion criteria and were randomly allocated to the pelubiprofen group (79 patients) or the celecoxib group (70 patients). Seventy-seven patients in the pelubiprofen group and 68 patients in the celecoxib group received at least 1 dose of study medication and were included in the safety population. Baseline characteristics of the two groups are summarized in Table 1. Because 2 patients in the pelubiprofen group and 1 patient in the celecoxib group dropped out without a primary end point data, 75 patients in the pelubiprofen group and 67 patients in the celecoxib group were included in the ITT population. Overall, 66 patients in the pelubiprofen group and 64 patients in the celecoxib group completed the study, and 62 patients in the pelubiprofen group and 58 patients in the celecoxib group, excluding protocol violators and patients with <80% drug compliance, were finally included in the PP analysis (Figure 1). No missing data was accommodated in the ITT analysis.

**Table 1:** **Baseline characteristics of patients with rheumatoid arthritis treated with pelubiprofen or celecoxib**

Variable	Pelubiprofen (n =77)	Celecoxib (n =68)
Age (mean ± SD, year)	54.3 ± 11.4	54.8 ± 10.8
Female sex, no. (%)	69 (89.6)	62 (91.2)
Disease duration (mean ± SD, month)	100.2 ± 104.2	89.8 ± 88.1
ACR functional class		
Class I, no. (%)	25 (32.5)	24 (35.3)
Class II, no. (%)	45 (58.4)	40 (58.8)
Class III, no. (%)	7 (9.1)	4 (5.9)
Class IV, no. (%)	0 (0.0)	0 (0.0)
DMARD use, no. (%)	55 (71.4)	47 (69.1)
Prednisolone use, no. (%)	54 (70.1)	42 (61.8)

ACR, American College of Rheumatology; DMARD, disease-modifying anti-rheumatic drug.

P values were determined using the Student t-test or the χ^2^ test.

### Efficacy profile

**Figure 2:**
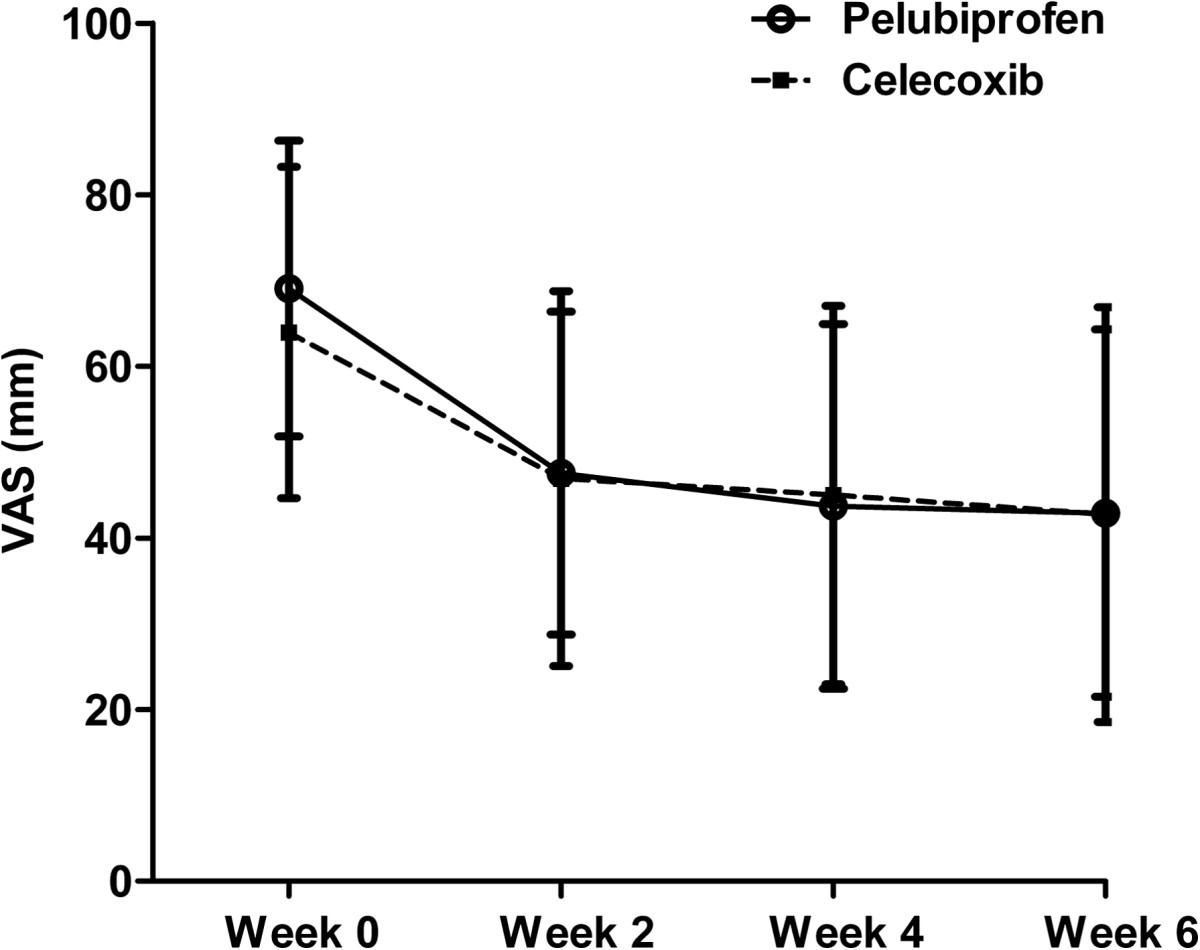
**Mean pain intensity in patients with rheumatoid arthritis treated with pelubiprofen 30 mg t.i.d. or celecoxib 200 mg b.i.d. as measured using a 100 mm visual analog scale (VAS).** Bars represent standard deviations.

Mean decrease ± SD of VAS pain was 26.2 ± 19.5 mm (range: 0.0-80.0 mm) in the pelubiprofen group and 21.2 ± 20.8 mm (range −20.0–90.0 mm) in the celecoxib group. The difference between two groups was 5.0 ± 20.1 and the lower limit of the 97.5% confidence interval was −2.3 mm, which was higher than the non-inferiority limit of −10.0 mm. Therefore, primary end point analysis indicated pelubiprofen was non-inferior to celecoxib (Figure 2). The results of ITT analysis for the primary endpoint were consistent with those of PP analysis (difference, 4.7 ± 20.8; 97.5% CI, −2.2 to ∞).

For the secondary end points, mean decrease in KHAQ was 0.2 ± 0.5 in the pelubiprofen group and 0.2 ± 0.5 in the celecoxib group (difference, 0.0 ± 0.5, 97.5% CI −0.2 to ∞). Median duration of morning stiffness was decreased by 0.0 minute in the pelubiprofen group and by 0.0 minute in the celecoxib group (p =0.99 by Wilcoxon’s rank sum test). Mean decrease in the frequency of rescue medication was −1.39 ± 3.68 in pelubiprofen group and −0.72 ± 3.39 in celecoxib group (difference, 0.7 ± 3.5, 97.5% CI −0.6 to ∞), and mean decrease in the total use of rescue medication was 1.38 ± 3.58 in pelubiprofen group and 0.71 ± 3.53 in celecoxib group (difference, 0.7 ± 3.6, 97.5% CI −0.6 to ∞) (Table 2). The results of ITT analysis for the secondary end point were consistent with those of PP analysis (data now shown).

**Table 2:** **Per protocol analysis of primary and secondary end points**

Variable	Pelubiprofen 30 mg (n =62)	Celecoxib 200 mg (n =58)	Difference	
			Mean (SD)	97.5% CI, one-sided
Patient’s pain VAS, 0–100 mm, mean (SD)				
Week 0	69.1 (17.2)	64.0 (19.3)		
Week 6	42.9 (21.4)	42.7 (24.2)		
Difference (Week 0 – Week 6)	26.2 (19.5)	21.2 (20.8)	5.0 (20.1)	−2.3, ∞
KHAQ, mean (SD)				
Week 0	1.0 (0.7)	1.0 (0.7)		
Week 6	0.8 (0.7)	0.8 (0.7)		
Difference (Week 0 – Week 6)	0.2 (0.5)	0.2 (0.5)	0.0 (0.5)	−0.2, ∞
Duration of morning stiffness, min, median				
Week 0	30.0	30.0		
Week 6	10.0	15.0		
Difference (Week 0 – Week 6)^*^	0.0	0.0		
Frequency of rescue medication, times in last two weeks, mean (SD)				
Week 0	2.8 (4.0)	2.2 (3.2)		
Week 6	1.4 (2.9)	1.5 (3.4)		
Difference (Week 0 – Week 6)	1.4 (3.7)	0.7 (3.4)	0.7 (3.5)	−0.6, ∞
Total dose of rescue medication, tablets in last two weeks, mean (SD)				
Week 0	3.0 (4.0)	2.2 (3.2)		
Week 6	1.6 (3.1)	1.5 (3.6)		
Difference (Week 0 – Week 6)	1.4 (3.6)	0.7 (3.5)	0.7 (3.6)	−0.6, ∞

SD, standard deviation; CI, confidence interval; VAS, visual analog scale; KHAQ, Korean health assessment questionnaire.

*p =0.99 by Wilcoxon’s rank sum test.

### Safety profile

Thirty-nine patients (50.6%) reported 62 AEs in the pelubiprofen group, and 25 patients (36.8%) reported 33 AEs in the celecoxib group (*p* =0.09, Table 3). Twenty-four patients (31.2%) in the pelubiprofen group and 14 patients (20.6%) in the celecoxib group experienced 51 ADRs (34 vs. 17 events, respectively, *p* =0.15, Table 4). The most common ADRs were abdominal pain [10/77 (13.0%) in the pelubiprofen group vs. 2/68 (2.9%) in the celecoxib group, *p* =0.03]. The frequency of gastrointestinal ADR was higher in the pelubiprofen group (20.8 % vs. 8.8%, *p* =0.045). One serious AE was reported in each group (right femoral neck fracture after trauma resulting in surgery in the pelubiprofen group and left knee tendon rupture resulting in surgery in the celecoxib group). Investigators reported both as being definitely not related to the study medication.

**Table 3:** **Adverse events that occurred during the study**

	Pelubiprofen (n =77)		Celecoxib (n =68)		
	Patients, n (%)	Events, n	Patients, n (%)	Events, n	***p***-value^*^
Total adverse events	39 (50.6)	62	25 (36.8)	33	0.09
Gastrointestinal	21 (27.3)	29	7 (10.3)	8	0.01
Anorexia	2 (2.6)	2	1 (1.47)	1	
Nausea	2 (2.6)	3	3 (4.41)	3	
Vomiting	1 (1.3)	1	1 (1.47)	1	
Indigestion	3 (3.9)	2	1 (1.47)	1	
Epigastric discomfort	4 (5.2)	4	0 (0.0)	0	
Abdominal pain	10 (13.0)	10	2 (2.94)	2	
Diarrhea	4 (5.19)	5	0 (0.0)	0	
Constipation	2 (2.6)	2	0 (0.0)	0	
Systemic	11 (14.3)	13	4 (5.9)	4	0.10
Facial edema	5 (6.49)	5	1 (1.5)	1	
Edema	4 (5.19)	5	1 (1.5)	1	
Chest discomfort	1 (1.3)	1	0 (0.0)	0	
Nipple pain	0 (0.0)	0	1 (1.5)	1	
Weight gain	0 (0.0)	0	1 (1.5)	1	
Pain	1 (1.3)	1	0 (0.0)	0	
Fatigue	1 (1.3)	1	0 (0.0)	0	
Anemia	0 (0.0)	0	1 (1.5)	1	0.29
Respiratory	6 (7.8)	6	4 (5.9)	4	0.65
Nervous system	2 (2.6)	2	4 (5.9)	6	0.32
Skin and appendage	3 (3.9)	4	1 (1.5)	1	0.37
Musculoskeletal	1 (1.3)	1	2 (2.9)	2	0.49
Hepatobiliary	1 (1.3)	1	1 (1.5)	2	0.92
Reproductive system	1 (1.3)	1	1 (1.5)	2	0.92
Cardiovascular	1 (1.3)	1	1 (1.5)	1	0.92
Infection	0 (0.0)	0	1 (1.5)	1	0.29
Eye	1 (1.3)	1	0 (0.0)	0	0.35
Urinary	1 (1.3)	1	0 (0.0)	0	0.35
Psychiatric	1 (1.3)	2	0 (0.0)	0	0.35
Peripheral vascular	0 (0.0)	0	1 (1.5)	1	0.29

*p-value by the chi-square test: difference between the proportions of patients that developed an adverse event.

**Table 4:** **Adverse drug reactions that occurred during the study**

	Pelubiprofen (n =77)		Celecoxib (n =68)		
	Patients, n (%)	Events, n	Patients, n (%)	Events, n	***p***-value*
Total number of patients	24 (31.2)	34	14 (20.6)	17	0.15
Gastrointestinal	16 (20.8)	20	6 (8.8)	6	0.045
Anorexia	2 (2.6)	2	1 (1.47)	1	
Nausea	1 (1.3)	2	2 (2.94)	2	
Indigestion	2 (2.6)	2	1 (1.47)	1	
Epigastric discomfort	2 (2.6)	2	0 (0.0)	0	
Abdominal pain	9 (11.7)	9	2 (2.94)	2	
Diarrhea	1 (1.3)	1	0 (0.0)	0	
Constipation	2 (2.6)	2	0 (0.0)	0	
Systemic	8 (10.4)	10	3 (4.4)	3	0.17
Facial edema	5 (6.49)	5	1 (1.5)	1	
Edema	3 (3.9)	4	1 (1.5)	1	
Weight gain	0 (0.0)	0	1 (1.5)	1	
Pain	1 (1.3)	1	0 (0.0)	0	
Respiratory	2 (2.6)	2	1 (1.5)	1	0.63
Nervous system	0 (0.0)	0	1 (1.5)	1	0.29
Skin and appendage	0 (0.0)	0	1 (1.5)	1	0.29
Hepatobiliary	1 (1.3)	1	1 (1.5)	2	0.49
Reproductive system	1 (1.3)	1	0 (0.0)	0	0.35
Cardiovascular	0 (0.0)	0	1 (1.5)	1	0.29
Infection	0 (0.0)	0	1 (1.5)	1	0.29
Peripheral vascular	0 (0.0)	0	1 (1.5)	1	0.29

*p-value by the chi-square test: difference between the proportions of patients that developed an adverse event.

## Discussions

In the present study, pelubiprofen and celecoxib were compared with respect to analgesic and anti-inflammatory effectiveness and safety profile. Pelubiprofen was found to be non-inferior to celecoxib in terms of pain reduction, improving quality of life, and reducing morning stiffness. The frequency and usage of rescue medication decreased in both groups.

However, although pelubiprofen was found to be non-inferior in terms of efficacy versus celecoxib, its safety profile was less favorable. Pelubiprofen has been previously reported to be better tolerated than aceclofenac and to present lower risks of peptic ulcers, bleeding, and abdominal pain in patients with back pain [14]. However, in the present study, it was found to have a poorer GI safety profile than celecoxib in patients with RA in terms of numbers of GI events and in terms of numbers of systemic and overall AEs, which were almost twice as frequent in the pelubiprofen group. Furthermore, this pattern was similar for adverse drug reactions. However, mean pain reduction tended to be greater in the pelubiprofen group (26.2 mm vs. 21.2 mm). Further study is required to confirm its superior efficacy versus celecoxib and other non-selective NSAIDs in terms of pain reduction.

The present study has some limitations that warrant consideration. First, the study was too short to allow meaningful evaluations of the long-term AEs associated with pelubiprofen, especially regarding cardiovascular events [15]. Accordingly, future studies are required to evaluate its long term effects. Second, this study was performed on a selected population and specific inclusion and exclusion criteria were applied, such as, no concomitant high dose steroids or biologic DMARDs, which are commonly used to treat moderate to severe RA. In addition, only patients demonstrating flare after discontinuation of an effective NSAID were included, which is not optimal for generalizability. Third, the non-inferior margin of a −10 mm difference for a change in VAS pain was rather large. However, the intergroup difference between mean changes in VAS pain was 5.0 ± 20.1 (97.5% CI, −2.3 to ∞), and accordingly, the primary outcome of this study could have been achieved even if a stricter standard had been employed.

In one multi-institution clinical study conducted by Shin et al. on patients with back pain, it was reported that the pain reduction afforded by pelubiprofen was not inferior to that of aceclofenac, which is a non-selective NSAID [14]. The present study is the first to report that the analgesic and anti-inflammatory effects of pelubiprofen are non-inferior to celecoxib in patients with chronic inflammatory arthritis. Furthermore, the frequency and usage of rescue medication also decreased similarly in our pelubiprofen and celecoxib groups.

## Conclusions

Pelubiprofen was found to be as effective as celecoxib for the pain reduction and for relieving stiffness in moderate to severe RA. The usages of rescue medication during the 6 week study period were decreased in both pelubiprofen and celecoxib groups. Furthermore, both treatments were generally well tolerated, although pelubiprofen showed a less favorable GI event profile. The results of this 6-week trial suggest that both drugs are effective and sufficiently safe to use in patients with moderate to severe RA.

## Authors’ original submitted files for images

Below are the links to the authors’ original submitted files for images. Authors’ original file for figure 1 Authors’ original file for figure 2
