# Supplementary figures and images for: Comparison of the efficacy and safety profiles of a pelubiprofen versus celecoxib in patients with rheumatoid arthritis: a 6-week, multicenter, randomized, double-blind, phase III, non-inferiority clinical trial
Source: BMC Musculoskelet Disord. 2014 Nov 18;15:375. doi: 10.1186/1471-2474-15-375 (PMC4247700; doi:10.1186/1471-2474-15-375)

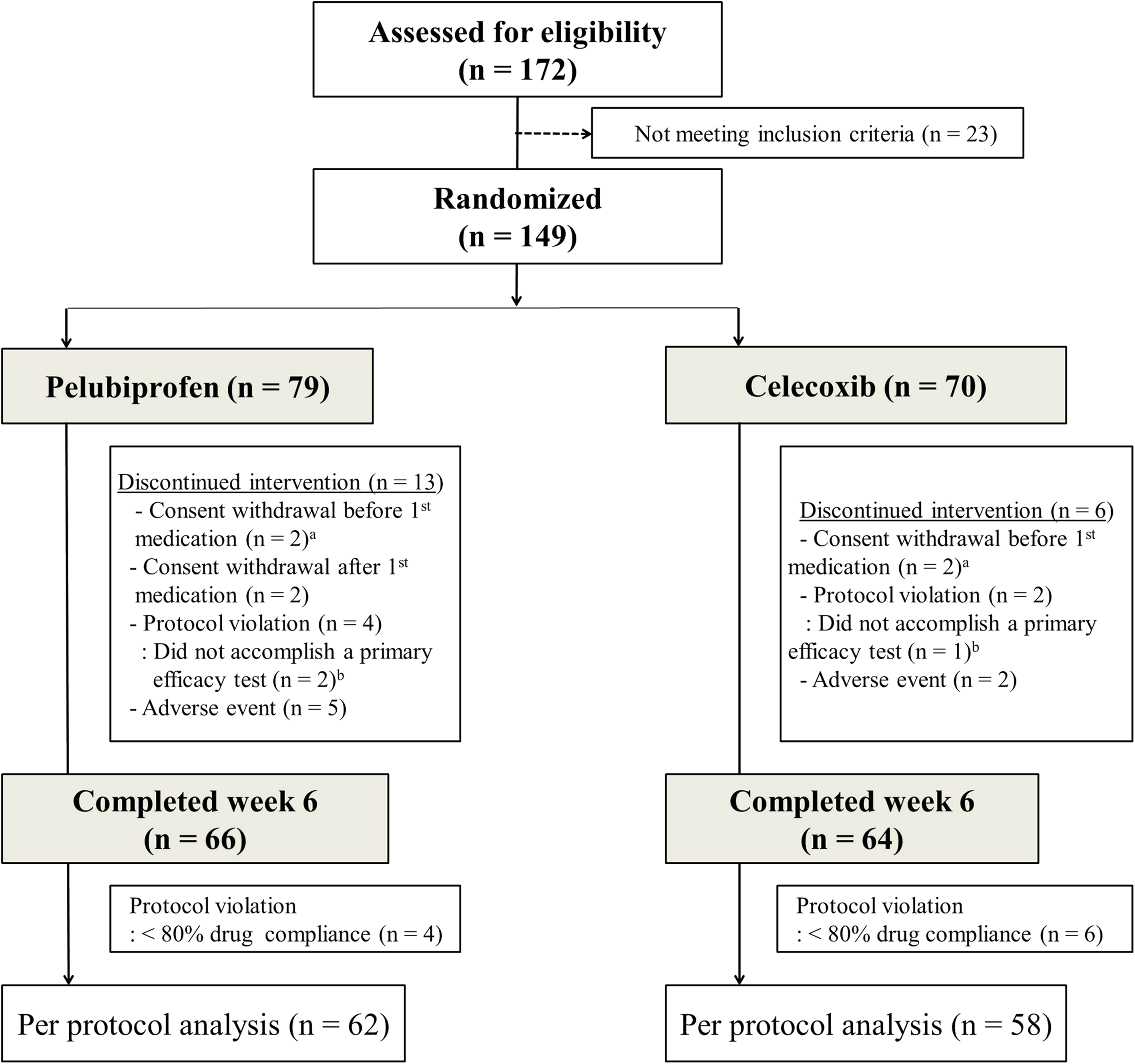

Supplement: Supplementary file 1 — Authors’ original file for figure 1 [file 12891_2014_2330_MOESM1_ESM.tif]

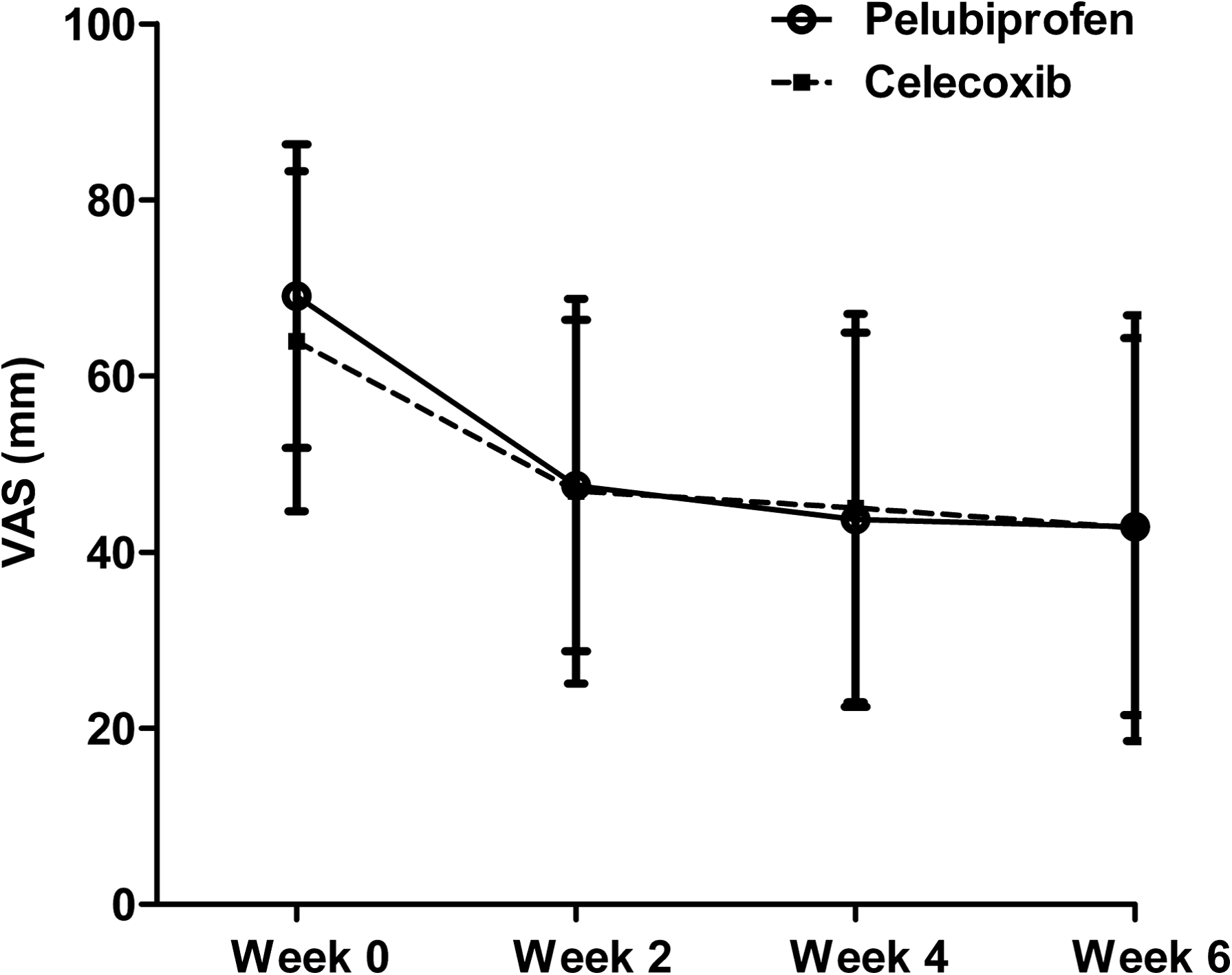

Supplement: Supplementary file 2 — Authors’ original file for figure 2 [file 12891_2014_2330_MOESM2_ESM.tif]
